# Advanced Artificial-Intelligence-Based Jiang Formula for Intraocular Lens Power in Congenital Ectopia Lentis

**DOI:** 10.1167/tvst.14.2.5

**Published:** 2025-02-04

**Authors:** Yan Liu, Xinyue Wang, Linghao Song, Yang Sun, Zexu Chen, Wannan Jia, Xin Shen, Yalei Wang, Xinyao Chen, Qiuyi Huo, Pranav Prakash Edavi, Tianhui Chen, Yongxiang Jiang

**Affiliations:** 1 Eye Institute and Department of Ophthalmology, Eye and ENT Hospital, Fudan University, Shanghai, China; 2 Key Laboratory of Myopia and Related Eye Diseases, NHC, Chinese Academy of Medical Sciences, Shanghai, China; 3 Shanghai Key Laboratory of Visual Impairment and Restoration, Shanghai, China; 4 Shanghai Medical College (SHMC), Fudan University, Shanghai, China

**Keywords:** congenital ectopia lentis (CEL), intraocular lens (IOL) power calculation, artificial intelligence (AI), prediction error (PE)

## Abstract

**Purpose:**

The purpose of this study was to develop an artificial intelligence (AI)-based intraocular lens (IOLs) power calculation formula for improving the accuracy of IOLs power calculations in patients with congenital ectopia lentis (CEL).

**Methods:**

A total of 651 eyes with CEL that underwent IOLs implantation surgery were included in this study. An AI-based ensemble formula—the Jiang Formula, was developed using a training dataset of 520 eyes and evaluated on a testing dataset of 131 eyes. A five-fold cross-validation and a two-layer ensemble learning model were constructed. The formula was then tested in a test set and compared against five current classic formulas.

**Results:**

The cohort included young patients (mean age = 14.38 ± 13.35 years). The Jiang Formula showed the lowest prediction error (PE; = 0.08 ± 1.01 diopters [D]), absolute error (AE; = 0.77 ± 0.65 D), median absolute error (MedAE; = 0.66 D), and root mean square error (RMSE; = 1.02 D) among six formulas (*P* < 0.001). Moreover, 68.00% of the eyes in the test set had AE within 1.0 D in the Jiang Formula.

**Conclusions:**

AI-integrated two-layer ensemble learning model demonstrates promising applications in IOLs power calculations for patients with CEL, not only providing higher predictive accuracy than current classic formulas but also accommodating extreme values and variations in surgical techniques.

**Translational Relevance:**

The Jiang Formula, an AI-integrated two-layer ensemble learning model, enhances IOLs power calculation accuracy in patients with CEL, ultimately improving surgical outcomes and supporting more effective, personalized treatment in this unique patient group.

## Introduction

Ectopia lentis (EL) is an ocular disorder where the lens, a critical refractive medium, is dislocated from its normal anatomic position.^1^ Due to inadequate support from the zonules, EL can result in significant refractive errors.^2^ Additionally, severe EL increases the risk of glaucoma, secondary microspherophakia, and corneal endothelial damage.^1^ EL usually can be a phenotypic feature of genetic disorders. The incidence of congenital ectopia lentis (CEL) is 6 per 100,000 people, with approximately 90% of affected individuals requiring intraocular lens (IOL) implantation surgery to improve vision.^3^ As refractive cataract surgery advances and with improvements in genetic testing and medical technology, both physicians and patients strive for accurate postoperative refraction prediction.^4^ Calculating the most accurate IOL power is crucial for optimizing patients’ visual outcomes.

The IOL power formulas have evolved to the fourth generation, achieving increasing accuracy in healthy eyes.^5^^–^^7^ However, there is a consensus that no single formula is universally optimal.^8^^–^^13^ The choice of the best formula often depends on the specific ocular characteristics and measurement.^14^ Research has indicated that patients with CEL typically have longer axial lengths (ALs), flatter corneas, and shallower anterior chambers. Menapace et al. identified preoperative AL as a significant factor contributing to errors in some IOL power calculation, with the median absolute error (MedAE) being lowest in long eyes.^15^^,^^16^ When selecting an appropriate formula for calculating IOL power, it is crucial to consider both AL and corneal refractive power, particularly in eyes with atypical biometric characteristics.^17^ Therefore, the accuracy is poor when applying existing formulas, primarily developed for Caucasian populations (with longer AL, deeper anterior chamber depth, and higher corneal curvature), to patients with CEL.^18^

Artificial intelligence (AI) has already been applied to the IOL power formulas, such as the Hill-RBF, Kane, and Pearl-DGS formulas.^19^^–^^21^ These AI-based formulas demonstrate significant advantages by incorporating additional ocular biometric parameters, constructing complex models, and utilizing large datasets through machine learning (ML).^10^^,^^22^^–^^24^ To improve the postoperative refractive accuracy for patients with CEL, this study included 651 CEL eyes, the largest known cohort in China.

We propose developing an AI-based two-layer ensemble learning model for calculating IOL power in CEL eyes. We used the Gradient Boosting Machine (GBM) and Multi-Layer Perceptron (MLP), ensemble learning models proven to outperform classical methods, to establish our Jiang Formula. We compare the Jiang Formula with five current classical formula—Barrett Universal II, SRK/T, Holladay 1, Hoffer Q, and Haigis—to evaluate its accuracy and stability.^25^ Through this comprehensive approach, we aim to improve the accuracy and applicability of refractive outcomes for this challenging patient cohort.

## Materials and Methods

### Data Collection

This study adhered to the principles of the Declaration of Helsinki and received ethical approval and informed consent authorization from the Human Research Ethics Committee of the Eye and ENT Hospital of Fudan University (ChiCTR2000039132). The research initially included patients with CEL, who underwent IOL implantation surgical treatment at the Eye and ENT Hospital of Fudan University, Shanghai, China, from March 2019 to June 2024. This clinical observational survey comprised both training/validation and test sets. All surgical procedures were performed by Dr. YX Jiang using a Centurion Vision System (Alcon Laboratories, Inc.). Details of the surgical techniques can be found in our previous publications.^2^^,^^26^^,^^27^ Patient demographics, including age, sex, gene mutation, and medical and surgical history, were documented. Preoperative biometric measurements, including AL, flattest and steepest keratometry (K1 and K2), anterior chamber depth (ACD), and white-to-white (WTW) distance, were recorded for all patients using an IOLMaster 700 (Carl Zeiss Meditec AG, Jena, Germany). The type and power of the IOLs implanted and postoperative refraction value were also collected. The types of IOLs implanted included AMO Tecnis ZCB00/PCB00, Tecnis Symfony ZXR00, Alcon SN60WF/SA60AT/SN6CWS, HumanOptic ASPIRA-aAY, Rayner 920H/970C, RAO600C, ZEISS SZ-1, and Hoya NY-60. Inclusion criteria for the study were: (1) successful biometric measurement with the IOLMaster 700; and (2) patients who underwent IOL implantation surgery. Exclusion criteria included: (1) history of corneal refractive surgery or ocular trauma; (2) requirement for additional surgeries due to recurrent fundus diseases; and (3) a less than 1 month interval between the surgery and postoperative refraction measurement.

### Refraction Prediction

**Figure 1.**
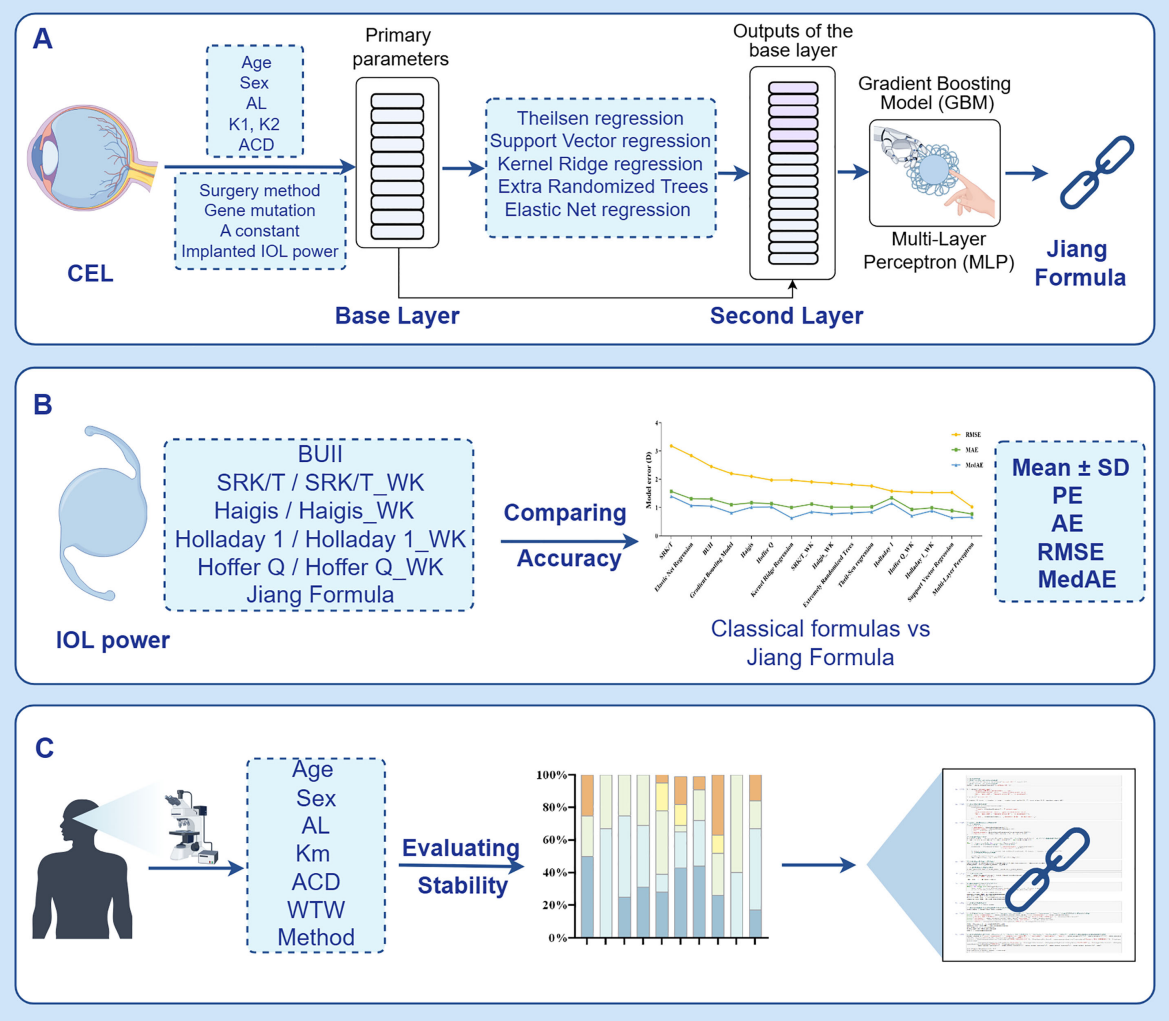
Flowchart of model development. (**A**) Demographic characteristics, ocular parameters, IOL information, and actual refraction of eyes with congenital ectopia lentis (CEL) were learned for training/validation, resulting in the Jiang Formula. (**B**) Accuracy comparison of the Jiang Formula and other classical formulas in the test set. (**C**) Evaluation of the Jiang Formula's stability and provision of a public access link. AL, axial length; ACD, anterior chamber depth; AE, absolute error; BUII, Barrett Universal II; CEL, congenital ectopia lentis; D, diopter; IOL, intraocular lens; Km, mean keratometry; MedAE, median absolute error; RMSE, root mean square error; PE, predicted error; SD, standard deviation; WTW, white-to-white.

Figure 1 illustrates the comprehensive methodology for developing and validating the AI-based formula—the Jiang Formula. During the development of the model, we performed model evaluation and selection through five-fold cross validation. The set of CEL eyes was randomly divided into the training/validation set (80%) and the test set (20%). Analysis involved eight features, including age, sex, AL, K1, K2, ACD, different gene mutations, and surgical methods (in-the-bag or out-of-the-bag).^28^^–^^30^ The actual postoperative refraction, expressed as spherical diopters (SD), was used as the training target.^31^ The model underwent iterative training with various feature combinations using a five-fold cross-validation for parameter optimization. A two-layer ensemble learning model was constructed to reduce overfitting, using a stacking strategy and nested cross-validation. In the initial preprocessing phase, raw data was converted into eight-dimensional features, with categorical attributes encoded as one-hot vectors and numerical attributes standardized. These processed features are independently trained across five models in the first layer, which include Theil-Sen regression, Support Vector Regression (SVR), Kernel Ridge Regression (KRR), Extremely Randomized Trees (ERT), and Elastic Net Regression. The second layer, included MLP and GBM, respectively, and integrated these outputs with original data features for outcome prediction. After comparison, the best-performing model was selected as the Jiang Formula.

### Formula’s Comparison

The accuracy and applicability of the Jiang Formula was evaluated against five classical formulas within the test set, using constants from the IOL Con website at https://iolcon.org. The formulas compared included: (1) Barrett Universal II (BUII, version 1.05, https://calc.apacrs.org/barrett_universal2105/); (2) Haigis (version 2004); (3) Hoffer-Q; (4) Holladay 1; and (5) SRK/T. Two-center optimized Wang-Koch (WK) adjustment was implemented for these formulas to refine prediction accuracy. Prediction performance was assessed based on several parameters: (1) mean and standard deviation of refractive prediction error (PE); (2) mean and standard deviation of absolute prediction error (AE), MedAE, and root mean square error (RMSE); (3) proportion of eyes within 0.50 diopters (D), 1.00 D, 1.50 D, and 2.00 D of AEs; and (4) impact of the original biometric parameters on formula stability.^32^ PE was defined as the difference between the spherical equivalent of postoperative manifest refraction and the formula-predicted refraction. A positive value indicated a hyperopic error, whereas a negative value indicated a myopic error. The absolute value of PE was referred to as the AE. The MedAE was used as the primary outcome due to the non-normal distribution of AE. The RMSE is another measure of the prediction accuracy, calculated as the square root of the average of the squared differences between the observed and predicted refractions.^12^

### Statistical Analysis

The normality of numerical data was evaluated using the Shapiro-Wilk test, with results presented as mean ± standard deviation. Categorical data were depicted as frequency percentages. The Wilcoxon rank sum test and the Chi-square test evaluated differences between the training and test set. The Kruskal-Wallis H test was used to evaluate the PEs of various formulas and models within the test set. Statistical significance was set at a *P* value of < 0.05. Statistical analyses were conducted using IBS SPSS Statistics 20 (version 20.0.1, 64-bit edition; IBM Corp.) and graphical representations were created using GraphPad Prism 8.

## Results

### Demographic and Clinical Characteristics

The characteristics of the total cohort, as well as the training and test sets, are presented in Table 1. A total of 651 eyes were included in the study. The average age of the patients was 14.38 ± 13.35 years. The mean AL was within the range of 24.82 ± 2.96 mm, and the mean keratometry (Km) was relatively low (41.07 ± 1.85 D). Comparisons between the training and test sets showed no significant differences in sex, age, AL, Km (mean K), ACD, WTW, IOLs power, actual postoperative refraction (standard deviation), and surgical methods.

**Table 1.** Demographic and Clinical Characteristics of Patients With Congenital Ectopia Lentis in the Training Set and the Test Set

Characteristics	Total Set	Training Set	Test Set	*P* Value
Patients, *n*	359	345	117	
Age at surgery, y	14.38 ± 13.35	14.57 ± 13.28	13.66 ± 13.63	0.677^a^
Sex, male/female	160/199	155/190	63/54	0.500^b^
Eyes, *n* (%)	651 (100)	520 (79.88)	131 (20.12)	
Preoperative parameter				
AL, mm	24.82 ± 2.96	24.93 ± 3.05	24.38 ± 2.57	0.122^a^
Km, D	41.07 ± 1.85	41.07 ± 1.86	41.08 ±1.81	0.866^a^
ACD, mm	3.22 ± 0.57	3.24 ± 0.58	3.17 ± 0.54	0.387^a^
WTW, mm	12.07 ± 0.56	12.06 ± 0.55	12.09 ± 0.58	0.590^a^
IOL power, D	22.55 ± 6.85	22.26 ± 6.90	23.48 ± 6.64	0.068^a^
Postoperative refraction, SD	−0.47 ± 2.37	−0.47 ± 2.46	−0.46 ± 1.99	0.928^a^
Surgery method				
In the bag, *n* (%)	497 (76.3)	401 (77.1)	96 (73.3)	0.356^b^
Out of the bag, *n* (%)	154 (23.7)	119 (22.9)	35 (26.7)	

ACD, anterior chamber depth; AL, axial length; IOLs, intraocular lens; Km, mean keratometry; SD, spherical diopters; WTW, whiter-to-white.

a Wilcoxon rank-sum test.

b Chi-square test.

### Accuracy Evaluation

**Figure 2.**
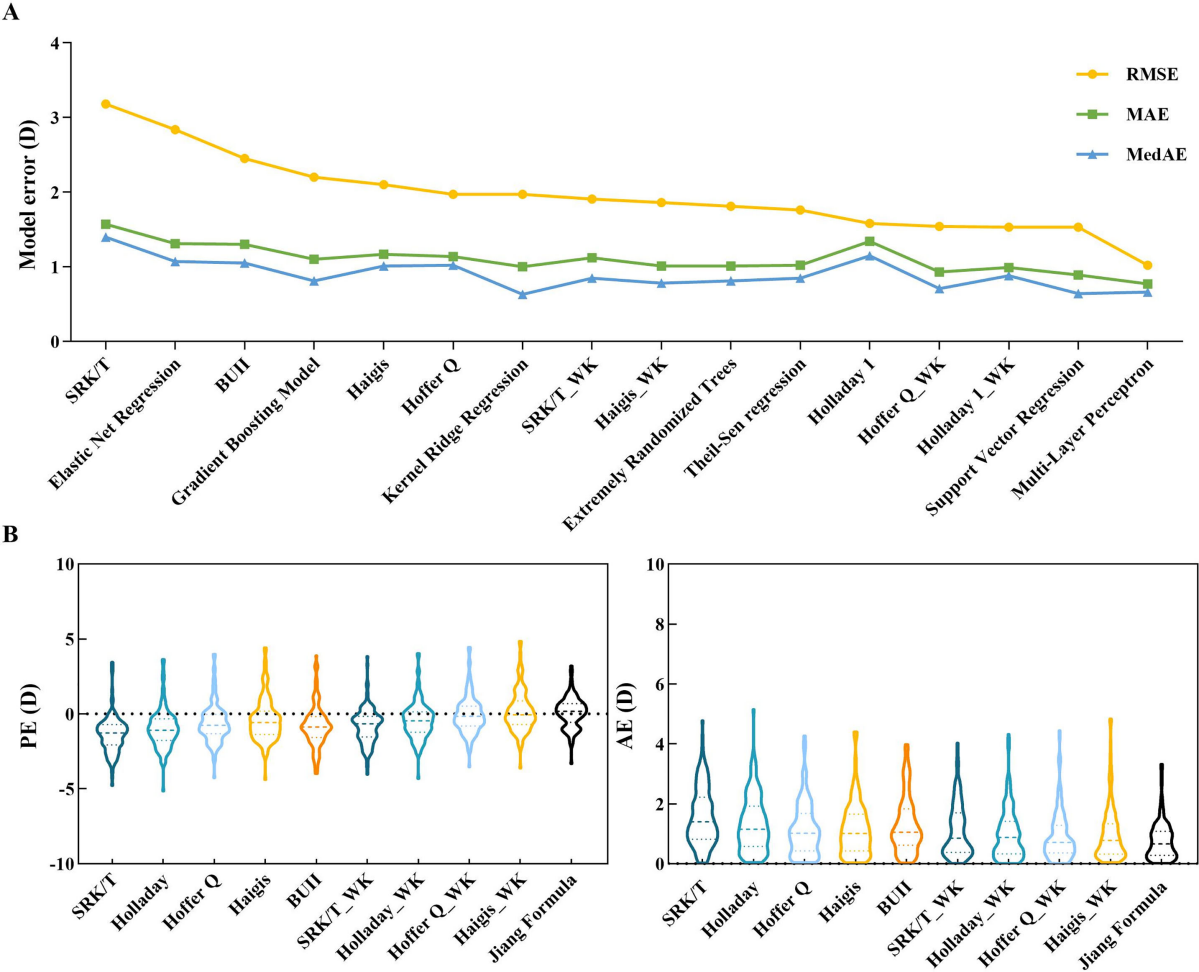
Comparison of the accuracy of the Jiang Formula with five classical formulas and six basic algorithms. (**A**) Performance of median absolute error (MedAE), mean absolute error (MAE); and root mean square error (RMSE). (**B**) Performance of predicted error (PE) and absolute error (AE). Classical formulas: Barrett Universal II, SRK/T, Holladay 1, Hoffer Q, Haigis. Basic Algorithms: Theil-Sen regression, Support Vector Regression (SVR), Kernel Ridge Regression (KRR), Extremely Randomized Trees (ERT), and Elastic Net Regression.

**Figure 3.**
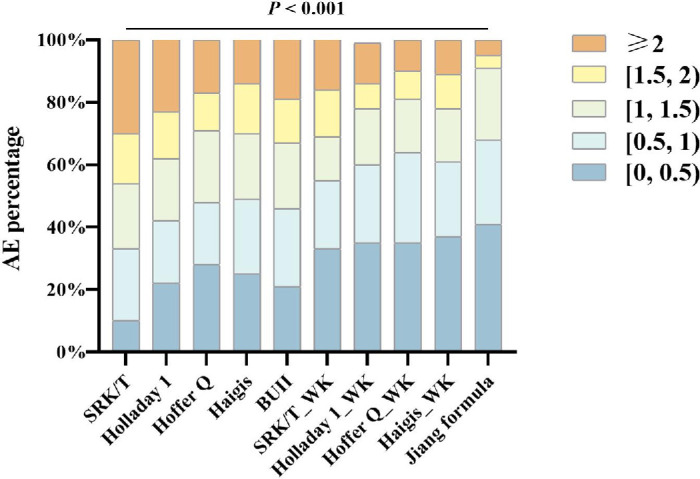
Proportion of absolute error (AE) within the ranges of 0.5 D, 1.0 D, 1.5 D, 2.0 D, and ≥2.0 D for the Jiang Formula and classic formulas.

Supplementary Table S1 presents the PE performance of each base algorithm, GDM, and MLP results. After comparison, the MLP integrated algorithm showed the best performance, which we refer to as the Jiang Formula. Figure 2A illustrates that the Jiang Formula outperformed the current classical formulas, demonstrating superior accuracy over any single basic algorithm. Table 2 demonstrates the prediction accuracy of the 10 IOL formulas in the test set. The Jiang Formula exhibited the lowest AE compared with the other 9 formulas (H = 215.62, *P* < 0.001). The order of AEs from lowest to highest was: Jiang Formula, Hoffer Q_WK, Holladay 1_WK, Haigis_WK, SRK/T_WK, Hoffer Q, Haigis, BUII, Holladay 1, and SRK/T (H = 83.01, *P* < 0.001). Additionally, the Jiang Formula had the lowest standard deviation of PE, at 1.01 D, with a mean PE of 0.08 D. It also showed the lowest MedAE and RMSE (0.66 D and 1.02 D, respectively). Figure 2B shows that both PE and AE results for the Jiang Formula were consistently closest to zero and the most stable. Figure 3 shows the proportion of AEs within 0.5 D, 1.0 D, 1.5 D, 2 D, and ≥2.0 D for the Jiang Formula compared with other classic formulas. The Jiang Formula had the highest proportion within the 1.0 D range, at 68.0%. Nearly half of the eyes (40.80%) were within the 0.5 D range in the Jiang Formula.

**Table 2.** Calculate Outcomes of the BUII, Haigis, Holladay 1, Hoffer Q, SRK/T, and Jiang Formula in Patients With Congenital Ectopia Lentis

Model	PE	*P* Value	AE	*P* Value	MedAE	RMSE
BUII	−0.80 ± 1.39	<0.001	1.30 ± 0.94	<0.001	1.05	2.45
Haigis	−0.61 ± 1.30		1.17 ± 0.90		1.01	2.10
Haigis_WK	0.17 ± 1.37		1.01 ± 0.93		0.78	1.86
Holladay 1	−0.98 ± 1.31		1.34 ± 0.93		1.15	1.58
Holladay 1_WK	−0.44 ± 1.23		0.99 ± 0.85		0.88	1.53
Hoffer Q	−0.61 ± 1.30		1.14 ± 0.87		1.02	1.97
Hoffer Q_WK	−0.07 ± 1.26		0.93 ± 0.84		0.71	1.54
SRK/T	−1.37 ± 1.24		1.57 ± 0.97		1.40	3.18
SRK/T_WK	−0.80 ± 1.37		1.12 ± 0.90		0.85	1.91
Jiang Formula	0.08 ± 1.01		0.77 ± 0.65		0.66	1.02

AE, absolute error; BUII, Barrett Universal II; MedAE, median absolute error; PE, predicted error; RMSE, root mean square error.

### Stability Evaluation

**Figure 4.**
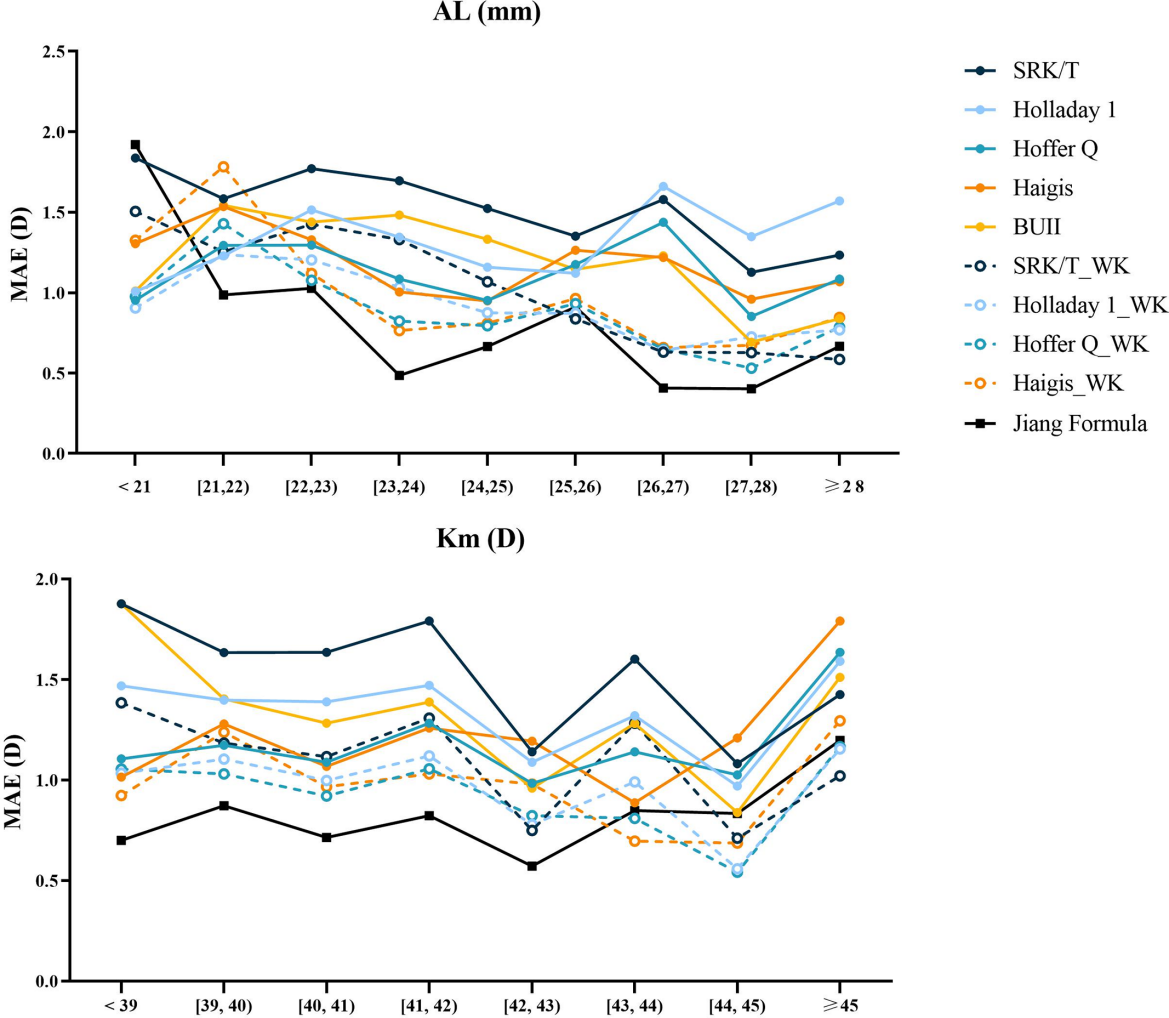
Median absolute error (MedAE) versus axial length (AL) and mean keratometry (Km). BUII, Barrett Universal II; D, diopter.

To evaluate the formula’s tolerance to variations in AL and Km, the mean absolute error (MAE) was categorized based on AL and Km, with the results depicted in line graphs in Figure 4. The Jiang Formula consistently maintained low and stable MAE values across different AL ranges. Notably, in groups with extremely long (AL ≥ 28 mm) AL, the Jiang Formula’s MAE was significantly lower than those of the other formulas. In eyes with AL between 21 and 28 mm, the Jiang Formula also exhibited the lowest error values. When categorized by Km, the Jiang Formula generally displayed the lowest MAE across various ranges. In the groups with the smallest Km values, the Jiang Formula’s MAE was slightly higher than that of the Haigis_WK formula.

**Figure 5.**
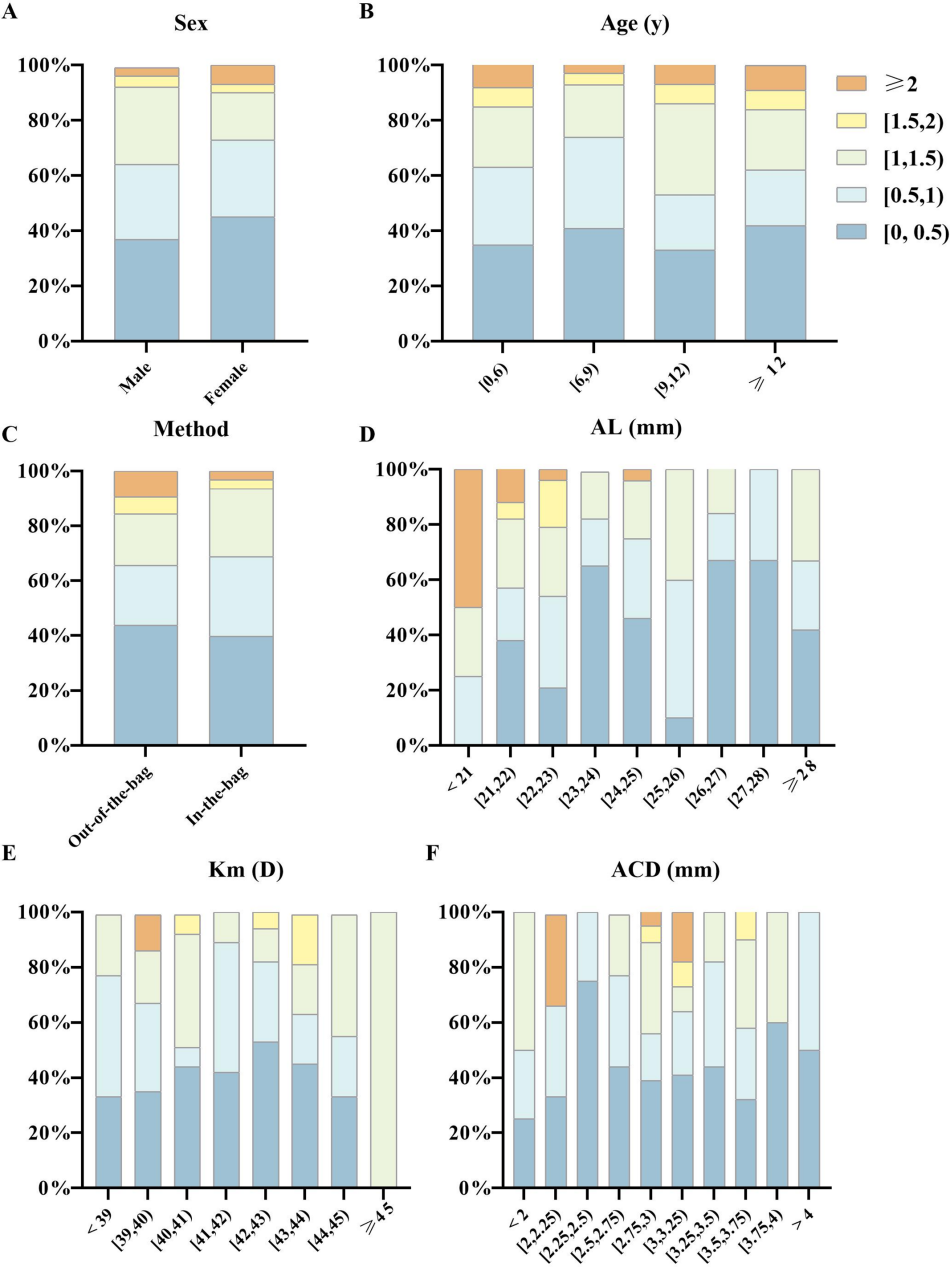
Proportion of eyes with absolute error (AE) in the ranges of 0.5 D, 1.0 D, 1.5 D, 2.0 D, and ≥ 2.0 D across different demographic and ocular biometric categories. (**A**) AE distribution by sex (male and female patients). (**B**) AE distribution by age group. (**C**) AE distribution by surgical approach. (**D**) AE distribution by axial length intervals. (**E**) AE distribution by mean keratometry intervals. (**F**) AE distribution by anterior chamber depth intervals. AL, axial length; ACD, anterior chamber depth; Km, mean keratometry; mm, millimeter; D, diopter.

The proportion of AE by surgical method, age, sex, AL, K1, K2, and ACD are shown in the histograms in Figure 5. The proportion of eyes with AE less than 1.0 D was higher in eyes undergoing in-the-bag IOL implantation compared with eyes with out-of-the-bag IOL. Across different AL categories, the proportion of AE showed no distinct pattern, with the proportion of AE less than 1.0 D consistently around 60%.

## Discussion

EL is a condition characterized by the displacement of the crystalline lens from its anatomic position due to dysfunction of the suspensory ligament, resulting in decreased visual acuity, myopia, or astigmatism in affected individuals.^33^ Untimely diagnosis and treatment can lead to severe amblyopia, particularly in children.^34^ Despite conservative management, approximately half of the patients with CEL ultimately progress to severe, permanent refractive amblyopia, making surgery the most reasonable option currently available.^35^^,^^36^ With the advent of precision medicine and the gradual increase in the 60-year survival rate of patients with CEL to 90%, there is an increased demand from both patients and surgeons for accurate postoperative visual quality and refractive predictions.^37^ In this study, through ML, we established an AI-based IOL refractive prediction model, termed the Jiang Formula, for patients with EL and compared its performance with that of single basic algorithms and five classical formulas, demonstrating superior accuracy and stability in all cases.

Current IOL power formulas applied in patients with CEL often yield significantly incorrect refractive outcomes, possibly because existing IOL power formulas are targeted at individuals with age-related cataracts.^38^ Despite the advancement of IOL power formulas to the fourth and fifth generations, patients with CEL have unique eye characteristics, such as corneal flattening and increased AL, which make fourth-generation formulas less accurate than the third-generation ones, with the SRK/T formula showing the highest accuracy.^5^^,^^25^^,^^28^^,^^38^ Previous research has shown that demographic characteristics such as age and gender, surgical approach, and preoperative ocular biometric parameters, particularly AL and corneal curvature, can influence the ideal IOL refractive prediction outcomes in patients with CEL.^39^ Although some studies have focused on selecting and optimizing IOL power formulas for patients with CEL, these variables do not occur in isolation and may be interrelated. Even with the SRK/T formula, the PE in patients with CEL still reach 1.289 D.^25^ Furthermore, this study innovatively incorporates the surgical approach as a contributing factor, facilitating a more personalized selection of IOLs power for patients with CEL to align with the goals of precision treatment.^36^^,^^40^ Therefore, there is a necessity for improving the accuracy of IOLs refractive prediction in patients with CEL.

Currently, AI-based formulas with or without baseline formulas have been widely applied in cataract and highly myopic individuals, with PEs significantly lower than those of classical formulas, and the proportion within the ±1.0 D range has been increased.^5^^,^^41^ ML, a subset of AI, can learn and recognize complex patterns from large datasets, allowing adaptation to various contributing factors, and making it more efficient than theoretical formulas. To our knowledge, we have the largest EL database in China and possibly globally. In this study, we abandoned baseline formulas and utilized AI to generate a novel formula. We selected five basic algorithm learners, including Theil-Sen regression, SVR, KRR, ERT, and Elastic Net Regression for the first stage of a stacking model based on their complexity and ability to capture different aspects of the data.^41^ For example, SVR is a kernel-based method capable of modeling complex nonlinear relationships. Elastic Net Regression combines the advantages of ridge regression and lasso regression, using regularization to avoid overfitting. The two-layer stacked ensemble learning model effectively combines and utilizes the strengths of these five basic algorithms, further improving prediction accuracy and stability, and then utilizes the results data from the first layer for regression supervised learning to predict or adjust the results for the second layer, ultimately achieving the desired outcomes.^5^ MLP is a feedforward artificial neural network capable of learning nonlinear models, with strong adaptability, widely used in classification, regression, feature learning, and other ML tasks.

In this study, we included five classical formulas for comparison, with PEs of IOL refractive power in patients with CEL distributed between 0 and 2 D, still falling short of the ideal refractive state. The Hoffer Q formula exhibited the lowest PE, which may be attributed to its inclusion of only AL and Km values. We observed that the predictive performance of the Jiang Formula obtained after the stacking regressor surpassed that of any single AI model or classical formula. The PE, AE, and RMSE values of the Jiang Formula were lower than those of classical formulas and any single basic algorithm. Furthermore, evaluation of the Jiang Formula based on age, gender, AL, Km, and ACD revealed that 60% of PE values fell within the ±1.0 D range, with good tolerance for extreme values. Therefore, a two-layer stacked ensemble learning model is feasible in developing the IOL power formulas, and the methods adopted in this study are standardized, extending their application to other databases. We have open-sourced the code and created an online demonstration website (https://github.com/Brickzhuantou/Jiang-Formula) that can calculate the IOL refractive power for patients with EL based on the Jiang Formula.

There are some limitations in the present study. First, the AI model is currently limited by a relatively small dataset and a lack of validation from external databases. Subsequent endeavors will prioritize the inclusion of additional data and actively seek validation from compatible external databases. Second, the growth of the pediatric eye poses challenges in selecting artificial lens power, and further validation and optimization of the Jiang Formula’s performance are needed.

In summary, the newly developed Jiang Formula presents a novel approach for determining IOL power in patients with CEL. Using a two-layer stacked ensemble learning approach, this model integrates five ML algorithms, showcasing superior performance compared to other classical formulas. Furthermore, it exhibits improved tolerance and accuracy in addressing cases with extreme AL and Km. Whereas optical-based methods remain essential, ML enhances performance for patients with CEL by uncovering patterns in historical data that may be missed by conventional techniques. This capability to identify hidden patterns provides a substantial clinical advantage, leading to more accurate IOL power predictions and better surgical outcomes for patients with complex ocular characteristics.

## Supplementary Material

Supplement 1
