# Supplementary material for: Advanced Artificial-Intelligence-Based Jiang Formula for Intraocular Lens Power in Congenital Ectopia Lentis
Source: Transl Vis Sci Technol. 2025 Feb 4;14(2):5. doi: 10.1167/tvst.14.2.5 (PMC11801393; doi:10.1167/tvst.14.2.5)
Supplement: Supplement 1 [file tvst-14-2-5_s001.pdf]

Supplementary table 1. Calculate outcomes of the 5 basic algorithms and 2 ensemble models in congenital ectopia lentis patients.

Model	PE	*p*	AE	*p*	Median AE	MSE
**Theil-Sen regression**	0.14 ± 1.32		1.02 ± 0.85		0.85	1.76
**Support Vector Regression**	0.27 ± 1.21		0.89 ± 0.89		0.64	1.53
**Kernel Ridge Regression**	0.15 ± 1.40	< 0.001	1.00 ± 0.99	< 0.001	0.63	1.97
**Extremely Randomized Trees**	0.16 ± 1.34		1.01 ± 0.90		0.81	1.81
**Elastic Net Regression**	0.18 ± 1.68		1.31 ± 1.07		1.07	2.84
**Gradient Boosting Model**	0.10 ± 1.49		1.10 ± 1.00		0.81	2.20
**Multi-Layer Perceptron**	0.08 ± 1.01		0.77 ± 0.65		0.66	1.02

AE, absolute error; BUII, Barrett Universal II; PE, predicted error; MedAE, median absolute error; MSE, root mean square error.
